# Facing the COVID-19 Pandemic and Developing a Sustainable Entrepreneurial Ecosystem: The Theory and Practice of Innovation and Entrepreneurship Policies in China

**DOI:** 10.3390/ijerph19148797

**Published:** 2022-07-20

**Authors:** Shu Meng, Xin Gao, Lianfeng Duan

**Affiliations:** 1 School of Business, Shantou University, Shantou 515063, China; mengshu@stu.edu.cn; 2 School of Business and Management, Jilin University, Changchun 130022, China; 3 Department of Chemistry, Shantou University, Shantou 515063, China; lfduan@stu.edu.cn

**Keywords:** COVID-19, sustainable development, entrepreneurship ecosystem, innovation and entrepreneurship policy

## Abstract

Under the influence of the COVID-19 pandemic, the vitality of start-ups has been continuously suppressed, their income has been decreasing, and overall economic development has gradually declined. At this time, the government, as an effective subject, should present its due responsibility to make entrepreneurship more sustainable and form a sustainable entrepreneurship ecology that can cope with risks. This paper takes the innovation policy theory and practice from research regarding China’s COVID-19 cases. One example is exploring the formation of the government’s innovation entrepreneurship policy and its mechanism within industrial cluster theory. Furthermore, we explore the analysis of the practice situation and try to solve the obstacles in the process of sustainable development through the regional entrepreneurial ecosystem and platform system building. We hope to find an acceptable way for the sustainable development of entrepreneurial ecological theory research and provide effective research and practical support.

## 1. Introduction

Innovation and entrepreneurship policies benefit from the comprehensive influence of various factors such as era change, cultural endowment, historical evolution, economic development among others [1]. From the perspective of era change, human beings have developed from the steam engine era during the first Industrial Revolution in the 1750s to the technologically advanced era of the 21st century, which is largely represented by the major innovation and integration of artificial intelligence, clean energy, digital technologies and the internet [2]. Large data storages, internet, cloud computing and digital technology promote innovation in many fields and have spawned a series of new business models as well as new businesses. Innovation and entrepreneurship gradually became globally recognized for their contributions to theory and industry [3]. From the perspective of cultural endowment, innovation is the inherent temperament of the Chinese nation. Chinese civilization has developed over 5000 years from the Chinese peoples’ constant efforts in self-improvement and innovation. For thousands of years, innovation has always been the most distinctive endowment of the Chinese nation, and it is considered the ideological source of the Chinese people’s pursuit of progress. Innovation is also the biggest driving force for China’s sustainable development. In terms of historical evolution, Chinese people first started with a rural household contract responsibility system. In order to reform the urban state-owned enterprises, Chinese government opened up the collective economy and developed the private economy. All these actions were aimed at mobilizing the enthusiasm and creativity of the Chinese people [4]. “New China” opened the door of self-reliance and independent innovation. A reformed and open market ignited the spark of entrepreneurship and innovation. From the perspective of economic development, China’s economy has shifted from high growth stage to high quality development stage; however, traditional growth momentum gradually weakened and due to resource and environmental constraints as well as gradual cost increases, the government must identify the economic growth point and implement supply-side structural reform as well as an innovation-driven development strategy to accelerate the conversion of new production factors (knowledge, technology, information, data) which would ultimately achieve rapid sustainable economic development [5]. Thus, innovation and entrepreneurship policies were introduced in China.

In order to take initiative in the new pattern of technological revolution and industrial transformation, China must rely on innovation [6]. Calls for innovation and entrepreneurship began with an executive meeting of The State Council in October 2013, emphasizing “mobilization of social capital forces to promote the growth of small and micro enterprises, especially innovative enterprises, to promote employment and promote the development of emerging productive forces”. Since the 18th National Congress of the Communist Party of China, General Secretary Xi Jinping has proposed to maximize the support and help of scientific and technological personnel in innovation and entrepreneurship. The world has limited resources and unlimited human potential. This is mass entrepreneurship and innovation on a wider scale and at a higher level. In this way, Li Keqiang issued a call for “mass entrepreneurship and innovation” at the Summer Davos Forum in September 2014. He called for a new wave of “mass entrepreneurship” and “grassroots entrepreneurship” on the area of 9. 6 million square kilometers of land, forming a new trend of “mass innovation” and “innovation for everyone”. Since then, he has frequently explained this key word in the first World Internet Conference, The State Council executive meeting, and various other occasions. In 2015, Premier Li Keqiang said in the work report of the government: “mass entrepreneurship and innovation”. In the government work report, promoting mass entrepreneurship and innovation “will not only expand employment and increase people’s income but also help promote vertical social mobility, fairness, and justice. “When talking about the culture of entrepreneurship and innovation, the report emphasizes that “people can better realize their spiritual pursuit and their own value in the process of creating wealth”. The government’s innovation and entrepreneurship policies (“mass entrepreneurship and innovation” policies) were issued accordingly. The general Office of the State Council agreed to promote mass entrepreneurship innovation via an inter-ministerial joint conference system for the development of spaces to promote public innovation and foster entrepreneurship guidance, for further employment entrepreneurship under the new situation, to deepen the reform of innovation entrepreneurship education. Regarding the promotion of the mass entrepreneurship innovation policies and measures, the State Council agreed to speed up the construction of mass entrepreneurship in order to provide support for a platform for the guidance of innovators and strengthen the national innovation-driven policy on development strategies [7]. However, under the influence of COVID-19, the vitality of start-ups has been greatly suppressed. The solution to this risk and restoration of the strong development of the economy as soon as possible is a key issue that scholars need to pay attention to.

## 2. Concepts Related to Innovation and Entrepreneurship Policies

### 2.1. Connotation and Metric

#### 2.1.1. Connotation of Innovation and Entrepreneurship

Entrepreneurship policy is a relevant policy today, and measures must be taken for the purposes of encouraging entrepreneurship and promoting the growth of start-ups through the improvement of the existing system, culture, and other environmental factors as well as fully utilizing policy instruments to promote entrepreneurial activities [8]. Innovation and entrepreneurship policy refers to the central and local government institutions, under the guidance of certain developmental concepts, that stimulate the innovation potential of the whole society and maintain entrepreneurial vitality in order to achieve mass entrepreneurship, innovation goals, and determine the code of conduct or action plans through a series of notices, opinions, letters, regulations, programs, regulations, and announcements. Innovation and entrepreneurship policies generally include researchers, college students, migrant workers, veterans, returned and foreign talents, and other innovation and entrepreneurship groups. The space generally includes, new areas at the state-level, development zones, science and technology innovation centers, mass entrepreneurship and innovation demonstration bases, and other functional zones. These structures generally include the introduction of technology and knowledge diffusion, research and development, manufacturing, product marketing, talent, operating capital, service environment, and other elements. Behavior generally includes innovation, development, service, entrepreneurship, management, construction, reform, investment, research and development, among other measures. In terms of the content, the instrument can be divided into specific measures such as science and technology planning, entrepreneurship, employment, tax incentives, investment and procurement, science and technology diplomacy, technology transfer, intellectual property rights, and science popularization, etc. [9,10,11].

#### 2.1.2. Measures of Innovation and Entrepreneurship Policies

The most common way to categorize a policy is to use policy instruments. There are five main categories of policy instruments. According to the classification of the areas of influence, the R–Z framework is divided into three categories: the supply-based policy instrument, environmental policy instrument, and demand-based policy instrument [12]. Classification according to the function and implementation process of the policy instruments are formed by Hood; policy instruments are divided into two categories: exploratory instruments and impact instruments [13], depending on the degree of direct participation of the government. In the H–R framework, policy instruments include: voluntary policy, mandatory policy, and mixed policy instrument [14]. According to the level of government intervention, the Doelen framework divides policy instruments into legal instruments, economic instruments, and communication instruments [15]. According to the way of government guidance, the S–I framework has policy instruments that include: authoritative instruments, inducement instruments, symbolic or persuasive policy instruments, capacity building symbols, and learning instruments [16].

So far, the R–Z framework is used widely and has the most mature system. However, the existing literature only distinguishes different types of policy tools, but it is not clear which categories are included in each tool. Therefore, the high-frequency words that meet the definition and have common characteristics were originally sorted out and summarized by means of coding and extraction. This paper intends to use the R–Z framework to determine the specific policy instruments involved in China’s mass entrepreneurship and innovation policies.

1. Supply-type policy instruments

Supply-type policy instruments refers to the government utilizing talent, information, technology, and capital support directly to expand the supply of technology, improve the supply of technology innovation related elements, and promote technological innovation and new product development. The instruments can be subdivided into education training and personnel training, information support, technical support, infrastructure construction, capital investment, public services, etc. Education training and personnel training mainly refers to the relevant government functional departments that establish long-term and comprehensive planning of talent development and actively improve the education and training systems at all levels, and attract overseas talents to serve the country according to the needs of industrial development. International talent exchange channels provide abundant technology innovation activities of different levels of human resources. Information support mainly refers to the government collecting and sorting out industrial and technical information at home and abroad and providing public information services for technological innovation activities through the construction of information infrastructure such as information networks, libraries, databases, and so on, so as to reduce and avoid information asymmetry in technological innovation. For example, “building a list of data resources of public institutions”, “formulating a catalogue of government data sharing and opening”, “coordinating the establishment of national information resource databases of population, legal entities, natural resources, spatial and spatial geography, and macro economy”. Technical support mainly refers to the government’s efforts in technical guidance and consulting to assist technological innovation in industry and strengthen the construction of technical infrastructure, including investing in research and laboratory development, establish learning mechanism, let state-owned research institutions engage in industry development related to technology and product development and spread to private enterprises, and take corresponding measures to encourage state-owned enterprises to take the lead in introducing foreign advanced technology. Infrastructure construction refers to the construction of existing platforms to build new infrastructure or improve various infrastructure construction. For example, the establishment of mass innovation spaces, entrepreneurship incubator platforms, innovation and entrepreneurship bases, science and technology parks, national independent innovation demonstration zones, research and development centers with universities, and technology centers with small and medium-sized enterprises. Financial support refers to the direct financial support provided by the government to enterprises for technological innovation such as providing research and development funds and infrastructure construction funds. Public service refers to the professional consulting service institutions where the government provides corresponding supporting service facilities, including transportation, communication, medical treatment, import and export, and related affairs, in order to ensure the smooth progress of technological innovation such as administrative supervision, intermediary services, consulting services, social services, etc.

2. Environmental-type policy instruments

Environmental policy instruments refers to the government’s intervention in financial regulation, tax systems, and other policies of environmental factors affecting the development of science and technology. The government provides a favorable policy for technological innovation and other technological activities for the environment, and thus, indirectly influences and promotes scientific and technological innovation and new product development. This intervention can be subdivided into target planning, financial support, tax incentives, intellectual property rights, regulations, etc. Target planning refers to the ability of the government to make the overall outline of the goal achievable by legitimate norms and legal procedures. This is not only the goal that the government expects to achieve, but also the goal of the relevant departments. Financial support mainly refers to the government to encourage enterprise innovation through financing, subsidies, venture capital, concessions, property allocation arrangements, equipment provision and services, loan guarantee, export credit loans, and other policies. Tax incentives mainly refer to the tax reduction and exemptions to enterprises and individuals including investment deduction, accelerated depreciation, tax exemption, and rent tax deduction. Intellectual property protection refers to the government’s clear division of property ownership of metadata and knowledge products formed after the use of data, to ensure that the interests of property owners are not infringed, which is also a policy means to promote the development of innovation and entrepreneurship. Regulation control refers to the government issuing management regulations and protection systems in order to standardize the behaviors of relevant subjects of innovation and entrepreneurship, so as to ensure that the introduction of innovation and entrepreneurship is carried out in a controllable, safe, orderly, and stable state. It includes “establishing and improving the government’s big data collection system”, “formulating systems and regulations for public data sharing as well as establishing an evaluation, assessment, and security review system for public data sharing and opening”, and “The Ministry of Information Industry and the State Administration of Quality and Technical Supervision formulate national standards for software products”. Strategic measures refer to the development of regional policies to encourage business mergers or alliances, and policies to encourage the introduction and innovation of technology with the goal of assisting industrial development. These can include, for example, building an innovation evaluation system and strengthening leadership; introducing policies to encourage the implementation of new technology and innovation including “software system analysts and system engineers with intermediate technical titles or above, or major inventions and creations, solving the identification of oneself and spouses and minor children”, “allowing technology patents and scientific and technological achievements to invest and giving the shares to inventors and contributors”, “establishing income distribution incentive mechanisms, encouraging enterprises to reward scientific and technological personnel with outstanding contributions”.

3. Demand-based policy instruments

Demand-based policy instruments refer to the government reducing market uncertainty through procurement and trade control measures, and actively exploring and stabilizing the market of new technology application so as to drive technological innovation and new product development, which can be divided into government procurement, consumer subsidies, outsourcing, trade control, overseas institutional management, and other aspects. Government procurement refers to the concept of providing a clear and stable market through the bulk procurement of new products, reducing the uncertainty faced by enterprises in the early stage of innovation, and stimulating the determination of enterprises to innovate. Government procurement includes central or local government procurement, public utilities procurement, etc. Consumer subsidy refers to the government subsidies for consumers on the demand side of the market, including subsidizing enterprises and innovative individuals to reduce the costs incurred in the process of innovation and entrepreneurship in order to further promote them. Outsourcing occurs when government agencies entrust research and development plans to enterprises or private research institutions to promote their research and development. Trade control mainly refers to the government control measures concerning import and export, including trade agreements, tariffs, monetary regulations, etc. Overseas institutional management refers to the management system in which the government directly or indirectly sets up data research and development institutions overseas and introduces corresponding management measures.

In general, supply-based policy instruments are seen as the driving force of policy for science and technological activities, demand-based policy instruments are manifested as the driving force, and environment-based policy instruments play an indirect influential role.

#### 2.1.3. Theoretical Review

Industrial cluster theory is a western economic theory that appeared in the 1980s. The theory of industrial cluster was founded by Michael Porter, an authoritative scholar in the field of competitive strategy and international competition at Harvard Business School in the 1980s. Industrial cluster theory comprises the gathering of a group of interconnected companies, suppliers, related industries and specialized systems and the association of clusters in this region to form effective market competition in a specific area of a special field, building specialized production elements to optimize cluster depressions, making the enterprise sharing areas public facilities, marketing environment and the external economy, all in order to reduce the costs associated with the exchange of information and logistics. This forms the regional agglomeration effect, scale effect, external effect, and regional competitiveness. Therefore, the gathering and governance of enterprises within the regional scope becomes very important, which is also the basic function of entrepreneurial ecology. Therefore, this paper also focuses on the mechanism of operation of the government as an important subject in the construction of entrepreneurial ecosystem, which is mainly generated through government policies and mainly applies the theory of industrial cluster.

### 2.2. Formation and Development

#### 2.2.1. Formation of Innovation and Entrepreneurship Policies

Gilbert, Audretsch, and McDougall (2004) [17] trace the emergence of U. S. startup policy. According to the industrial organization framework [18], industrial or market performance is determined by the underlying industrial structure. This market structure is in turn influenced by the importance of the key factors used by the industry. Market demand, market structure, market performance, and government response within the framework together constitute the important factors affecting the development of the industry. When the superior efficiency of mass production within the industry over small businesses became more apparent, a political debate emerged on how to solve the small business problem. Therefore, the development of small enterprises has attracted wide attention, and globalization policies, technology development policies, and supporting policies have gradually formed from local to regional to state to federal level. Subsequently, it was adopted and used for reference in Europe, Asia, and Germany. The article points out that entrepreneurship policy is likely to become the most important policy instrument for the global knowledge-based economy [19].

#### 2.2.2. Innovation and Entrepreneurship Policy Development

1. Entrepreneurship policy for overseas returnees

Chen et al. (2010) [20] pointed out that the key to the transition from a development-oriented government to a service-oriented government is to provide equal and universal policy services. At present, some service-oriented local governments are actually still performing the functions of developmental governments. In the case of Shanghai, Suzhou, and Wuxi, we analyzed the reasons and performance of these local governments in providing “nanny-style services” and point out that their government is still a developing government. Then, by studying the internal constraints of its development-oriented governance and the deficiencies of “nanny-oriented service”, it reveals the dynamic transformation trajectory of the local government from a development-oriented government to a service-oriented government, and it advocates that the role of government should be changed into a service-oriented government.

Gu (2015) [21], after analyzing and evaluating the overseas talent introduction policies, developed and implemented compensation and income tax, and an entrepreneurship support and housing subsidy from research funding. The government should gradually change from a development government to a service government, focusing on the actual needs of regional development, the organic integration of overseas talent and international technology cooperation, service and management of the introduction of overseas talent, and creating a good environment for innovation and entrepreneurship.

2. Entrepreneurship policy for college students

Pang and Ding (2014) [22] proposed that the national intellectual property system, commercialization policy, university entrepreneurship policy, resources, and culture can promote the creation of derivative enterprises. The support of university incubators and the operation and management mode of derivative enterprises are crucial to the success of enterprises. Yuan and Zhao (2016) [23] systematically combed and summarized Beijing college students’ entrepreneurship policies and deeply analyzed the main problems existing in the current policy environment, such as preferential policies to be implemented, financing environment, supporting services to be deepened, risk mechanisms to be improved among other issues, and put forward suggestions for development.

From the view of scholars, universities also need an incentive policy support from personnel, capital, and patented technology among other aspects to ensure coordination among policies. Various measures should be taken to attract and retain outstanding talents, provide sufficient research funds, and encourage scientists to use infrastructure to transform their research and development achievements. University research and development activities should be closely connected with the market, and they must pay attention to the transformation of emerging technological achievements with strong applications and high market matching degree.

## 3. Mechanism of Action and Evaluation Method of Innovation and Entrepreneurship Policies

### 3.1. Mechanism of Action

#### 3.1.1. Impact of Policies on the Formation of New Enterprises

Technological initiatives promote the development of the resource endowment needed for the entrepreneurial infrastructure. In particular, technological initiatives that encourage research and development (R & D) have broad impacts on new technologies, risks, and knowledge. Research shows a link between enterprise formation and the development of new technology [23] as well as university development [24]. Research shows that R & D spillover is particularly important in knowledge-intensive industries as it is for forming a cluster to cultivate new firms [25] by increasing labor mobility, university research awareness and regional industrial diversity, and ultimately accelerating the diffusion of new technologies [26]. Such broad initiatives can help build national-level resource endowments, such as new knowledge and a trained workforce, and increase the institutional arrangements and legitimacy of such activities in the eyes of newborn entrepreneurs, thereby promoting new enterprises for the technology.

Economic initiatives can take various forms and contribute to corporate infrastructure in many ways. First, they may include attempting to stimulate the economic environment by providing financial resources to a unique field of technology. For example, Pennsylvania’s nanotechnology program began in 1999 with a goal to stimulate the regional economy by providing direct funding to companies working in nanotechnology. Second, economic initiatives can promote cooperation between different parties, hold social events, and advocate for legislation. For example, the Texas Nanotechnology Program was launched in 2001 to bring together members of the industrial, academic, and government sectors in the field. In addition, economic initiatives support the establishment of institutions with legal, normative, and standardized technologies. Another example is the Colorado Nanotechnology Program that was initiated by academic and industry leaders to advocate for nanotechnology in the state. In these ways, economic initiatives fund the development of institutions and resources needed for the spiritual infrastructure of enterprise from which new businesses arise. Audretsch et al. (2015) [27] believes that a country’s technological initiatives and economic initiatives will help build the entrepreneurial infrastructure needed for new companies and that economic initiatives are positively related to the establishment of early-stage nanotechnology companies and the growth of nanotechnology companies in the region. Bardzell et al. (2017) [28] also proposed the same view, arguing that economic science and technology policies play an important role in the formation of new enterprises. Baradwaj et al. (2015) [29] found that federal funding support had a significant negative impact on entrepreneurial activities. However, it is generally believed that financial support policies can create a better business environment and encourage entrepreneurs to take entrepreneurial risks and that financial support can reduce their debt and cost pressure, increase their ability to obtain resources, and thereby increase the number of entrepreneurs.

#### 3.1.2. Impact of Innovation and Entrepreneurship Policies on Entrepreneurial Output

Direct government investment plans are venture capital funds created directly through the government. One of the most important projects identified by Fang et al. (2018) [30] is the need for government funds to work with private venture capital funds. It is also important in the market for areas where government funds have obvious and identifiable corporate financing failures, as structural barriers in the market lead to a relative lack of capital. The structure of government funds helps to minimize the agency costs associated with the financing of small and high-tech companies. For example, for fund managers, contracts controlling investment requirements and compensation incentives are useful for all invested companies, and they perform very well at reducing issues in private limited partnership venture capital funds, thus effectively helping companies acquire venture capital [31,32].

Preferential tax policies can promote the increase of incubator performance through the following mechanisms: firstly, establishing the national strategic position and function of the local government strengthens the support for the incubator and the investment to strengthen the value of the incubators. Therefore, the preferential tax policies of incubators have a significant incentive effect on the number of professional and technical personnel of incubator funds and incubators [33].

The migration of enterprises is affected by various policies of local or central government, but also by the availability and cost of production factors, the acquisition and maintenance of competitiveness, emotional sustenance, and personal preferences. Among the four factors affecting policy, economy, strategy, and emotion, government policy factors have the most significant impact on the migration of resource-based enterprises. For developed regions, more stringent environmental control policy has a positive significance in promoting the relocation of local resource-based enterprises. To attract resource-based enterprises in areas requiring more assistance, governments should improve their administrative efficiency while providing government facilities, a fair playing environment, as well as land and tax incentives [34,35].

Government subsidy policies have a significant positive impact on the relationship between entrepreneurial valuation, financing, management, market performance, and growth performance. Pergelova and Angulo-Ruiz (2014) [36] found that government funding support promotes growth in new ventures, noting that their performance is largely dependent on an entrepreneurs’ ability to mobilize existing business resources, and that policy makers should use policy instruments to help new ventures build a competitive advantage in the market. Government funding incentives have a significant negative impact on the sales growth of start-ups. Financial support policy can create a better business environment, encourage entrepreneurs to take entrepreneurial risks and pursue more business profits, thus improving performance; at the same time, financial support can increase the ability of entrepreneurs to obtain resources. Namely, government funding can be used by entrepreneurs to leverage other resources such as human capital, and research and development capital, so as to help entrepreneurs solve obstacles such as entry costs and capital difficulties and achieve better performance [37]. However, Eckhard and Shane (2003) [38] argue that policymakers should stop subsidizing start-ups and instead focus on supporting a small number of new companies with high growth potential.

### 3.2. Evaluation Method

Scholars have studied the methods of policy formulation and evaluation and put forward policy evaluation indicators such as policy satisfaction, and also proposed the MOS and other evaluation models to evaluate policy development status. McCann and Ortega-Argiles (2016) [39] of the MILES framework and SMEs marks a significant shift in the EU and is crucial to fostering entrepreneurship. The article suggests that policy resources must prioritize those with the most realistic opportunities to develop broad and large-scale areas of activities, technologies or sectors that also exist in many different local and interregional links [40].

#### 3.2.1. Evaluation of the Formulation of Innovation and Entrepreneurship Policies

Lundstrom and Stevenson (2001) [41] proposed the MOS model of entrepreneurship policy framework, which is the combination of motivation, opportunity and skill, so that policies for entrepreneurship can be designed and considered around these three elements: to motivate entrepreneurship at the individual level; to acquire the knowledge and skills; and to provide resources and environmental support for potential entrepreneurs. Henrekson and Sanandaji (2018) [42] proposed that the lack of incentives for stock options leads to tax issues according to the European innovation policy. Europe continues to lag behind the US in venture capital (VC) activities and the creation of successful new ventures and has recently been overtaken by China. Due to higher venture capital activity in countries with lower stock option tax rates, there is also more corporate activity with high growth expectations. The low tax rate on employee stock options further increases the relative return on entrepreneurial work and investment and transfers financial capital and talent to the field. Therefore, the article believes that a more relaxed tax on the proceeds of employee stock options can be a strategy for European countries to catch up with entrepreneurial financing. Cumming et al. (2009) [43] proposed that international entrepreneurship policies need to distinguish whether to support all companies or focus on the minority that are considered more likely to succeed in international efforts. The policy’s objective may be to promote new businesses with the potential for rapid international growth [44]. However, Fernhaber and Mcdougall Covin (2009) [45] et al. suggest that inexperienced teams may benefit the most from additional external knowledge. For businesses with international potential but entrepreneurs who lack the necessary expertise, the goal of the policy’s support may be to acquire foreign market opportunity identification skills and develop overseas contact networks through alliance partners and venture capital firms. The core of the demand-side innovation policy lies in providing the necessary legitimacy basis for the development path of the technology-driven innovation mode. Therefore, for China, it is the “best practice” to define the demand by itself and regain the confidence to discover and solve the problem of innovation incentives and diffusion, which is the urgency that is needed to learn in China [46].

#### 3.2.2. Evaluation of the Effects of the Implementation of Innovation and Entrepreneurship Policies

Wiklund et al. (2009) [47] uses goal-setting theory to analyze how and why entrepreneurs think that policies to promote academic entrepreneurship are effective. A joint study and data from 3136 assessments of 98 academic entrepreneurs showed that obtaining funding for a policy program is critical and enhanced their awareness of other policy measures, such as providing knowledge for access to non-financial resources (networks, business) and reducing administrative burden. A large number of scholars have raised questions regarding the lack of effectiveness of the government’s public policy for businesses [48,49], stemming from the argument that the policy itself is problematic. These policies cause people engaged in a marginal business to most likely fail with very little economic impact [50,51,52,53]. Additionally, there were not a lot of jobs created, which can possibly be attributed to generation and development of policy [54,55]. Therefore, Arshed et al. (2019) [56] conducted research on the formulation and development of the policy itself by opening the black box for the formulation process, using institutional theory for analysis and research to explore the participation of multiple participants in the institution to find out the root causes of policy failure in a specific institutional context in order to ensure the effectiveness of the policy.

Cheng and Cui (2016) [33] evaluated the policy transmission effect—the policy effect from the policy and the support benefit of the incubator—and proposed a multi-layer conduction incubator policy within the performance evaluation framework, and adopted the 2008–2012 incubator tax preferential policy through the implementation of research data: the three-stage joint cube equation model and social network services. Wan (2013) [57] put forward the structural equation model of entrepreneurship policy attraction, satisfaction, and loyalty to evaluate the effect of the implementation of the policy by setting the indicators of policy attraction, satisfaction, and loyalty. It is proposed that the relationship between attraction (pre), satisfaction (during), loyalty (post), and the influencing factors of entrepreneurship policies should be analyzed so as to determine the direction of policy formulation and provide support for policy evaluation. Huang et al. (2021) [58] put forward the “three-dimensional one” college students’ entrepreneurship policy evaluation model, using content analysis from entrepreneurs’ growth stage, business policy, and three levels of comprehensive analysis and evaluation, they built the six dimensions of entrepreneurship policy system. The system aims to promote entrepreneurial culture policy, entrepreneurship education policy, reduce barriers to entry, provide start-up capital support, provide business support and special group support, and improves upon the college students’ entrepreneurship policy evaluation theory. Piwowar-Sulej et al. (2021) [59] uses content analysis and the quantitative analysis method to analyze environmental policy text as a sample, and reveals that based on the perspective of policy instruments combined with the policy cycle dimension of the three-dimensional policy analysis framework, the framework can be used as a guide to analyze the rationality and effectiveness of sample policy.

## 4. Development Status of China’s Innovation and Entrepreneurship Policies

According to the Chinese government network “double gen” policy database of public data, as of 31 December 2021, the State Council and various ministries issued 265 innovation entrepreneurship policies (except approval notice) which include policies involving the State Council and the National Development and Reform Commission, Ministry of Science and Technology, Ministry of Education, Ministry of Finance, finance, human resources, the Ministry of Agriculture, Ministry of Commerce, culture, China, audit, taxation, industry and commerce, and the China banking regulatory commission. The first policy is the Outline of the Development of Agricultural Science and Technology (2001–2010) issued by the Ministry of Science and Technology and the Ministry of Agriculture in 2001. The policy is mainly concentrated from 2012 to 2019, rising rapidly year by year since 2012, reaching the highest in 2015, and decreased slightly decreased this year, showing an overall inverted U-shaped distribution.

### 4.1. The Context behind Landmark Policy Development

Since China’s implementation of the national long-term science and technology development plan (2006–2020), the construction of technology innovation systems made positive progress, but its ability to face Chinese enterprise innovation is still weak. Many areas lack independent intellectual property rights, and enterprises have not become part the decision regarding innovation, research and development, research organization, or application of the main body. Therefore, the General Office of the State Council’s main body issued a policy in 2013 to strengthen the innovation of enterprise technologies, comprehensively improve the innovation ability of the enterprises, establish research and development institutions, cultivate small and medium enterprises, and promote the industrialization of major scientific and technological achievements as the main tasks to improve the innovation ability of the enterprises. Within a target of two years, the policy hoped to introduce a basic formation with the market oriented enterprise as the main body using the combination of technology innovation systems.

In 2014, the state further focused on national economic and technological development zones, taking on the role of reforming the field and open pioneering in order to strive for the development of concepts, establishment, and management modes to speed up the transformation. These efforts to achieve the pursuit of quality from the pursuit of speed by the government lead the homogeneous competition to differentiation from the hard environment to soft environment. In 2015, entrepreneurial innovation was booming thanks to the internet. There was now a supporting platform for public entrepreneurship which was rapidly developing. This new platform stimulated in the people infinite wisdom and creativity, sped up the integration between the network economy and real economy, uses domestic and international innovation resources, improved production efficiency, and fostered new economic growth points. In 2016, the state set up innovation demonstration bases to promote models and typical experiences of mass entrepreneurship and innovation that adapt to different regional characteristics, organizational forms, and stages of development. The government set up the Beijing haidian district, Tianjin binhai new district central business district, Hangzhou Hangzhou future technology city 17 regional demonstration base, Tsinghua university, Shanghai Jiaotong university, and four other universities and research institutes demonstration base. They also set up the Haier group, Alibaba groupm and other seven enterprise demonstration bases to further promote the development of new technology, new products, new forms, new models, and provide support for the development of new kinetic energy. In 2017, the country strengthened the implementation of innovation-driven development strategy by broadening financing channels, promoted the transformation and upgrade of the real economy to further implement the “Internet +”, “made in China 2025”, a new generation of artificial intelligence and other major measures, focus on strengthening the construction of innovation entrepreneurship platform, foster emerging forms, develop the sharing economy, to promote China’s economy to maintain rapid growth, towards the high-end level to provide strong support. China’s economy, from a stage of rapid growth to a stage of high-quality development, has put forward higher requirements for innovation and entrepreneurship. In 2018, the country proposed to build “double gen” upgrade in a commitment to solve the innovation entrepreneurship ecology. The transformation mechanism of cientific and technological achievements is not perfect and the financing of large and medium-sized enterprises is not enough. The international cooperation for innovation of entrepreneurship and part of the policy implementation does not reach the designated position, and strives to achieve entrepreneurial employment, high quality innovation entrepreneurship clusters, international and domestic innovation resources depth, etc. In 2019, the state put forward the strategy of innovation-driven development and entered a new stage of development, and China’s low-cost advantage in the international arena gradually disappeared. Compared with the low-cost advantage, technological innovation has the outstanding characteristics of being not easy to imitate and high added value, so the established innovation advantage has a long duration and strong competitiveness. Implementing the strategy of innovation-driven development and accelerating the transformation from low-cost advantage to innovation advantage can provide a strong driving force for China’s sustainable development. Since then, the entrepreneurship policy with innovation-driven policy as the main strategy has been gradually implemented. (source: National Innovation and Entrepreneurship Policy Information Service Network).

### 4.2. Regional Policy Concerns

**Figure 1:**
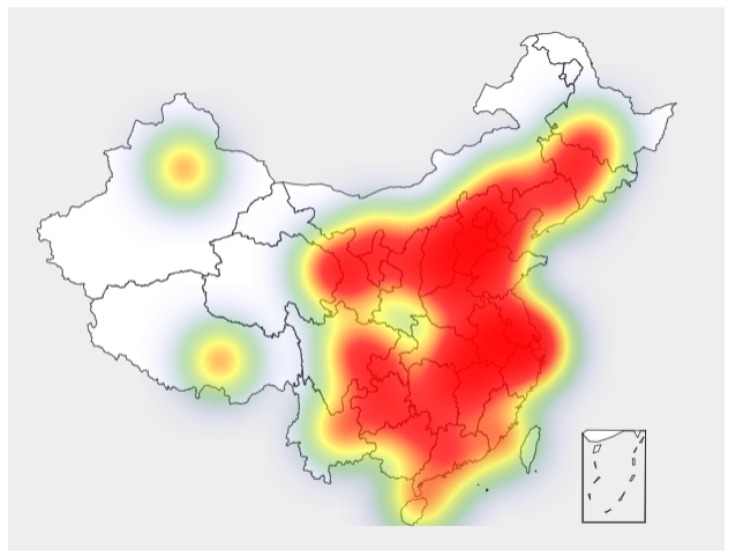
Distribution of regional policy quantity.

**Figure 2:**
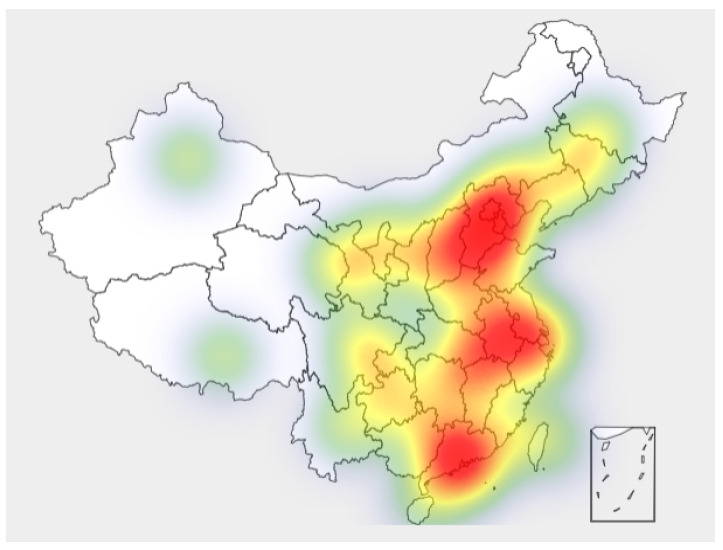
Distribution of regional policy attention.

Regional policies are also emerging, and the quantity and distribution of policies and regional attention are shown in Figure 1 and Figure 2 (data source: National Innovation and Entrepreneurship Policy Information Service Network, June 2022)

As can be seen from the Figure, innovation and entrepreneurship policies have basically covered most parts of China, among which the central region and southeast China have the largest number of policies, indicating that China’s innovation and entrepreneurship policies have been paid attention to in the region. The highest policy concerns were in Hebei, Anhui, Guangdong and its surrounding areas. At present, many innovation and entrepreneurship agglomeration zones, such as Zhongguancun, Hebei Baoding National High-tech Industrial Development Zone, Xiamen Software Park, and Shenzhen Chuangye Bay, are concentrated in areas with high policy attention. The increasing vitality of innovation and entrepreneurship has played an important role in adding new growth drivers, promoting economic development, expanding employment, and improving people’s living standards.

### 4.3. Effect of Innovation and Entrepreneurship Policies

**Figure 3:**
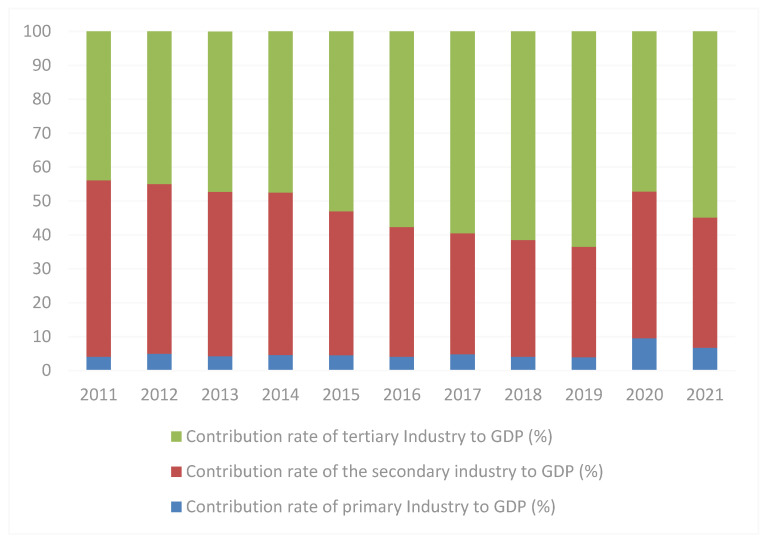
GDP contribution rate of the three major industries from 2011 to 2021.

According to the implementation of the policy, China has set up regional demonstration bases in universities, research institutes, and enterprise demonstration bases. Under the leading role of the demonstration base, China’s industrial upgrading has achieved remarkable results. Innovation and entrepreneurship have brought about the innovation of production technology, and technological innovation has caused changes in the industrial structure. Overall, the industrial structure of the whole industry has gradually evolved from the dominant proportion of the primary industry to the secondary industry and the tertiary industry. In 2015, the contribution rate of the tertiary industry was more than 50%, and continued to increase in recent years, increasing to 63.5% in 2019, but after facing the impact of the new outbreak, it reduced to 54.9% in 2021 (Japan and other developed economies growth mainly from consumption, the tertiary industry accounted for GDP at around 70% over the years), meaning that China’s economic structure is undergoing major changes, and that transformation and upgrading has reached a critical stage (Figure 3, Source: National Bureau of Statistics).

### 4.4. Policy Outputs around COVID-19

In the face of COVID-19, China has advocated for the use of fiscal tools to support the real economy. First, we will increase the deficit-to-GDP ratio and ease the contradiction between income and expenditure. The impact of COVID-19 on economic growth has slowed down, and continued tax and fee cuts have resulted in significant short-term reductions in government revenue. However, spending on epidemic prevention and control and livelihood protection has increased, and there is a serious contradiction between government revenue and expenditure. In particular, there is great pressure on community-level social security services, such as agriculture and social security. Therefore, it is necessary to appropriately increase the deficit-to-GDP ratio. Secondly, the issuance of special government bonds to support the fight against the epidemic is vital. The timing of the issuance of national debt is flexible and does not include the fiscal deficit. It can better respond to changes in the world economic situation and facilitate decision-making. In addition, the issuance is flexible and can be placed in a short period of time in one step to raise funds quickly, which brings convenient macro control. Third, we will increase the issuance of special bonds and expand nongovernmental investment. The use of special government bonds as project capital has greatly alleviated the financing difficulties of major local projects. In the face of the impact of the epidemic, the state has used a lot of support and assistance to deal with the impact of the epidemic. This unique pattern works effectively in China, given the government’s dominant role in regulating the market.

### 4.5. Looking for International

**Figure 4:**
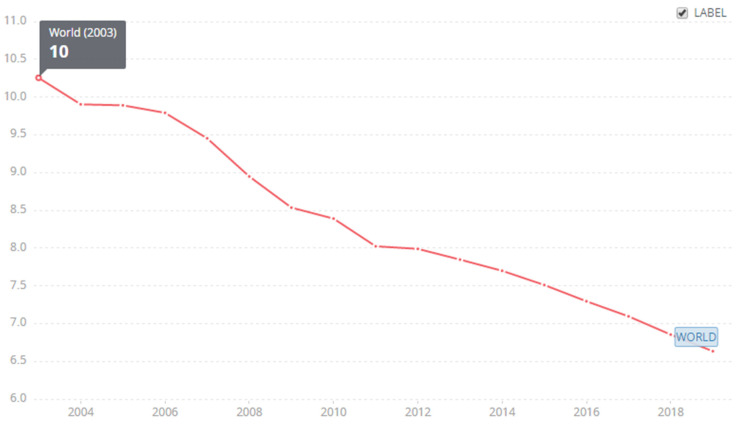
Start-up procedures to register a business (number).

In the international context, we can also get such strong support, that is, the procedures of registered enterprises are gradually simplified. Procedures tend to be simple according to Creative Commons Attribution 4.0 (CC-BY 4.0). This is precisely because of the important role of good environmental policy tools. As of 2019, the international level is 6.5, but China has reached the level of 5, which is lower than the average standard of international entrepreneurship’s process complexity. The government has made important efforts in this process (Figure 4, Source: The World Bank).

## 5. Future Research

### 5.1. Exploring the Action Mechanism of Demand-Based Policy Instruments with the Market as the Main Body

As an effective way to improve the efficiency of the use of demand policy instruments, innovation vouchers are a new form of government procurement, which has been widely used in the UK, Canada, Australia, and other developed countries or regions to improve the innovation level of the market [60], which is especially needed in the current situation of the COVID-19 pandemic. The basic principle of innovation vouchers is that the government issues innovation vouchers to enterprises, and enterprises use innovation vouchers to purchase scientific research services from research and development personnel, and research and development personnel hold the innovation vouchers to the government financial departments. Innovation vouchers can effectively solve the innovation needs of small and medium-sized enterprises. The advantage is that they can only buy technological innovation services, rather than use them as cash, to ensure the special use of special funds, and improve the efficiency of the use of funds. Innovation vouchers reduce the innovation investment cost for small and medium-sized enterprises, increase the technical service income for universities and research institutes, and build a market bridge between small and medium-sized enterprises, universities, and research institutes. The implementation of the innovation voucher system is combined with the improvement of enterprise innovation ability, optimizing industrial policies, and promoting the rapid growth of R & D investment in society [61]. It is an important means to improve the conversion rate of achievements in innovation and promote the combination of industry, universities, and research. Therefore, as an innovative form of government procurement, innovation vouchers are a feasible choice in the policy instrument to promote the development of innovation in small and medium-sized enterprises. In the future, we should consider and improve the specific use rules, procedures, and specifications of innovation vouchers. In addition, the government’s energy is limited, making it difficult to invest all the human, financial and material resources to build infrastructure such as data open platforms. Outsourcing this technical work to enterprises with sufficient technology can not only reduce the pressure on the government, but also enhance social support for government work. Policy details of outsourcing should also be considered in the future. According to Lundstrom and Stevenson’s interpretation of entrepreneurship policy, it is necessary to promote the motivation, opportunities, and skills of individual entrepreneurs, and it is necessary to explore the path of demand-based policy tools to promote the motivation, opportunities, and skills of individual entrepreneurs.

### 5.2. Exploring the Relevant Research on the Mechanism of Government Action Based on Financial Support

According to this policy, the Chinese government is constantly expanding financial support channels and encouraging investment in small and medium-sized enterprises, but such an investment is risky, so despite the government’s repeated calls for investment in small and medium-sized enterprises, the effect is not significant. China has established local financial institutions, mainly urban commercial banks, which are an important component and special group of China’s banking industry. Its predecessor was urban credit cooperatives established in the 1980s. Its business positioning at that time was to provide financial support for small and medium-sized enterprises and pave the way for the local economy. Since local financial institutions are close to small and medium-sized enterprises, they are very familiar with the production and operation of enterprises and the character of enterprise operators, which to some extent can make up for the disadvantage of insufficient review materials. Due to sufficient enterprise information, they are more willing to provide loans to small and medium-sized enterprises.

In practice, the urban commercial banks with good operating performance are mainly concentrated in those economically developed areas, especially in the eastern regions. Its main performance depends on local government revenue, less negative impact on urban commercial banks, small and medium private enterprises, strong profitability, urban commercial banks strong willingness to provide loans to small and medium-sized enterprises, high per capita income, developed credit culture, and local governments’ high awareness of protection of private property rights. However, as is the case with most urban commercial banks, the main problem is that the market positioning is not clear. Many city commercial banks as well as state-owned banks and joint-stock commercial banks prioritize big customers and big projects and can’t give small and medium-sized enterprises and citizens a full range of quality services. Secondly, China also has special policy financial institutions for small and medium-sized enterprises, such as China development bank for small and medium-sized enterprises. Policy-based financial institutions play two roles: one is to supplement private financial institutions to provide policy loans to small and medium-sized enterprises; the other acts as policy instruments to divide their funds fairly and reasonably, so that every SME can be a potential beneficiary and maintain the fairness of policies. For example, Japan loans government emergency aid funds to SMEs through the platform of policy-based financial institutions, and expands the amount of the guaranteed loans for ICBC and policy investment banks, which indirectly enlarges the loan funds to SMEs. However, from a practical point of view, the loans and investment of China Development Bank mostly target large and medium-sized capital construction projects and key enterprises in infrastructure, basic industries, and pillar industries. It is difficult for small and medium-sized enterprises to apply for China Development Bank loans, and the access threshold is high. China Development Bank requires enterprises to provide perfect financial statements and collateral, but small and medium-sized enterprises generally have no perfect financial system and cannot provide statements. In addition, small and medium-sized enterprises themselves also lack collateral.

To sum up, although China has urban commercial banks for small and medium-sized enterprises, and policy financial institutions for small and medium-sized enterprises, there are still great difficulties in the implementation of the policy, and small and medium-sized enterprises still face the dilemma of “financing difficulties”. Therefore, the method of implementation of the policy is particularly critical [62]. The government should establish and improve the government-led credit guarantee system. A credit guarantee system, on the one hand, can solve the problem of small and medium-sized enterprises by playing a role in supporting the development of small and medium-sized enterprises; on the other hand, it can create a safe credit environment for financial institutions, fully mobilizing the enthusiasm of all kinds of financial institutions for small and medium-sized enterprises, but also can control the credit loan risk. Finally, the credit system can guarantee national financial funds to be fair and reasonably distributed to small and medium-sized enterprises. The government’s funds to support small and medium-sized enterprises are not given to a specific enterprise, but to each SME through the credit guarantee system platform, thus maintaining the fairness of the policy. At present, China’s credit guarantee institutions are mainly private guarantee companies, with few government guarantee institutions. Private guarantee companies, especially those guarantee companies with single business variety, low personnel quality, and limited risk management level are difficult to engage with in business. Government financing guarantee institutions play a small role in increasing credit for loans to small and micro businesses. From the perspective of building a credit system for micro, small, and medium-sized enterprises, China is still in its infancy. From the cities where the system was established, i.e., in Suzhou, Shenzhen et al., describes the existing problems including (1) a slow update of the enterprise’s credit system. On the one hand, the system is increasingly incompatible with the existing line system of various departments. It is difficult for all departments to submit information through the network platform in a timely fashion; on the other hand, there is also a lack of a strong leadership. Many departments using the line system to submit information to the personnel changes. There is formation of “no successor”: line information data is submitted to the corresponding stagnation state; (2) Institutions, systems, and personnel cannot be fully integrated. The government has not equipped the corresponding personnel to reprocess the information. (3) The existing model of the existing enterprise credit system lacks market sustainability. For the public credit information service systems, the collection of credit file information mainly comes from the People’s Bank of China and various government departments. It has a strong color of government information disclosure and sharing promoted by local government administration. Information is limited to the basic information and public credit information of enterprises, especially the actual commercial credit information of small and micro enterprises that have never had loans with banks, such as purchase and sale situation, cash flow, and other information collection is basically in a state of absence, but this information can best reflect the actual credit level of the majority of small and micro enterprises. Therefore, it is particularly important to establish and improve the government-led credit system. In the future, the government should vigorously build a government-led credit guarantee institution. In establishing the credit guarantee system for small and medium-sized enterprises, it should increase the number of published data, merge and control the decentralized database, determine the standard system of data opening, ensure the supply of personnel for information reprocessing, and reduce the waste of social resources.

### 5.3. Exploring the Important Impact of the Supervision of New Industries, New Models and New Forms of Business on the Development of Enterprises

With the development of Internet technology, the importance of information is increasing. Information is related to the development of enterprises and even national security. Therefore, China should pay more attention to the Internet, information technology and network security, as well as strengthen the supervision of new industries, new business forms and new models, and increase the frequency of regulatory policy instruments [63]. While maintaining a relatively loose regulatory environment for the new economy, we should reflect more on the principle of prudence and even strict discipline by strengthening the supervision of new industries, new business forms and new models, especially via the supervision of personal privacy, security and other issues, so as to reduce the occurrence of adverse events. According to Chinese law, information security can be protected by a number of legal rights such as copyright Law (copyright Law), trade secret rights, privacy rights, personal rights, contractual claims, and anti-unfair competition protection under different circumstances. However, the lack of laws and regulations related to data disclosure, data security, and data property rights has become the primary obstacle affecting the healthy development of the big data industry. The government should introduce relevant laws on the protection of intellectual property rights of big data as soon as possible, and clearly define the rights and responsibilities of the collection, storage, transaction, transmission, and secondary utilization of data and information. To improve the relevant provisions of the Tort Liability Law, we should establish a set of data within the tort liability legal system that organically combines civil liability, administrative liability and criminal liability.

### 5.4. Focus on Building a Sustainable Entrepreneurial Ecosystem

The entrepreneurial ecosystem is symbiotic and self-maintained [64]. The diversity of the entrepreneurial ecosystem is reflected in its many participants, the creation of common values based on a common goal of chasing opportunities, the self-sustenance and self-enhancement in the regional environment depending on features such as the location, existing culture, etc. China’s existing entrepreneurial ecosystem policies for the regional entrepreneurial ecosystem advocates the introduction of a variety of entrepreneurial subjects, including enterprises, in order to provide services for entrepreneurial agencies. This actually led fierce competition within the entrepreneurial ecosystem and disturbed the entrepreneurial ecosystem’s homogeneity. China’s policies reflect the diversity, network- type, and competitiveness of the entrepreneurial ecosystem. However, China’s mass entrepreneurship and innovation policy does not pay attention to whether there is a common vision between enterprises, nor does it pay attention to the self-maintenance and strengthening function of the entrepreneurial ecosystem, nor the establishment of different entrepreneurial ecosystems according to the actual situation of local regions.

The government should pay attention to cultivating the cultural atmosphere of the entrepreneurial ecosystem and build a common vision of the entrepreneurial ecosystem [65]. The government needs to encourage social organizations to hold entrepreneurship training activities, entrepreneurship and innovation competitions, and various public welfare activities centered around building mass entrepreneurship and innovation to cultivate a maker culture and create a strong atmosphere of “mass entrepreneurship and innovation”. At the same time, the government should not only establish entrepreneurship parks and bases, but also build a bridge for entrepreneurship parks, guide service docking, improve supporting service measures, build a good regional and platform ecosystem, and promote the overall development of innovation and entrepreneurship. Government departments at all levels should connect the entrepreneurship park with research institutions, universities, law institutes, industry associations, leading enterprises, social organizations, and encourage research institutions to open cooperative research, academic exchanges and talent training to improve innovation ability and technology as well as guide scientific and technological achievements. Governments must encourage cooperation between entrepreneurship parks and universities to promote employment. They must encourage industry leaders to enter the entrepreneurship park, share experiences, and provide assistance. Additionally, through technical guidance and consulting to assist the innovation of industry technologies and strengthen the construction of technical infrastructure, such as the development of laboratories, establish learning mechanism, let state-owned research institutions engage in the research and development of products in industry-development related technologies that can spread to private enterprises, and take corresponding measures to encourage state-owned enterprises to introduce foreign advanced technology to build a good ecosystem, further promoting the development of innovation and entrepreneurship. Considering the influence of the region, the government should encourage local governments to combine their own economic, geographical, and cultural advantages, and draw on the experience of the achievements of other entrepreneurial ecosystems.

In addition, the entrepreneurial ecosystem not only includes the regional entrepreneurship ecosystem, but also the platform entrepreneurship ecosystem [66]. Platform ecosystem is built on a platform of interconnected suppliers, distributors, and developers. The competitiveness of platform ecosystems depends on member enterprises to improve their performance through the platform, especially in developing more valuable products for end users. The core of the platform ecosystem is large enterprises, such as the Alibaba Group with Alibaba as the core, and the Xiaomi ecological chain with Xiaomi as the core. The growth of enterprises into platform enterprises realizes the transformation of business model from “single win” to “multi-win”, while the growth of platform enterprises into platform ecosystem has achieved the “win-win” effect. The platform ecosystem is changing all walks of life, and has penetrated all aspects of people’s lives, promoting the rapid development of the whole social economy [67,68]. Therefore, in the future, China’s policies should also encourage large enterprises to build platforms, create a platform ecosystem, and form a sustainable and stable entrepreneurial ecosystem to cope with the many risks brought by COVID-19.

## 6. Conclusions

We conclude that, in the context of COVID-19, countries and companies should address the many risks posed by the pandemic and make extensive efforts to build a government-led entrepreneurial ecosystem to form a sustainable and efficient circular economy. In order to implement a circular economy through the entrepreneurship ecosystem operation, we must pay attention to cost and capital elements at the same time in addition to the heart of the configuration of the possibility and rationality of the matter and energy circulation link. Through the entrepreneurial ecosystem, the effective integration of ecological material, energy, time, space, capital, and other elements of the ecosystem of sustainable development of businesses will be realized.
